# Caves as microrefugia: Pleistocene phylogeography of the troglophilic North American scorpion *Pseudouroctonus reddelli*

**DOI:** 10.1186/1471-2148-14-9

**Published:** 2014-01-16

**Authors:** Robert W Bryson, Lorenzo Prendini, Warren E Savary, Peter B Pearman

**Affiliations:** 1 Department of Biology and Burke Museum of Natural History and Culture, University of Washington, Box 351800, Seattle, WA 98195-1800, USA; 2 Division of Invertebrate Zoology, American Museum of Natural History, Central Park West at 79th Street, New York, NY 10024-5192, USA; 3 Department of Entomology, California Academy of Sciences, 55 Music Concourse Drive, Golden Gate Park, San Francisco, CA 94118, USA; 4 Landscape Dynamics Unit, Swiss Federal Research Institute WSL, Zuercherstrasse 111, Birmensdorf CH-8903, Switzerland

**Keywords:** Last Glacial Maximum, Refugia, Species distribution model, Scorpiones, Vaejovidae

## Abstract

**Background:**

Survival in microrefugia represents an important paradigm in phylogeography for explaining rapid postglacial re-colonization by species in temperate regions. Microrefugia may allow populations to persist in areas where the climatic conditions on the surface have become unfavourable. Caves generally contain stable microclimates and may represent microrefugia for species capable of exploiting both cave and surface habitats (troglophiles). We examine the phylogeography of the troglophilic North American vaejovid scorpion *Pseudouroctonus reddelli* using 1,993 base pairs of mitochondrial and nuclear DNA sequence data generated from 12 populations. We use (i) descriptive measures of genetic diversity and population genetics statistics, (ii) reconstructions of phylogeographical structure, spatial diffusion during diversification, and population sizes through time, and (iii) species distribution modelling to test predictions of the hypothesis that caves serve as microrefugia. We compare phylogeographical patterns in *P. reddelli* with other troglophilic species across the Edwards Plateau karst region of Texas.

**Results:**

Results revealed high haplotype and nucleotide diversity and substantial phylogeographical structure, probably generated during the Pleistocene. Spatial diffusion occurred along the southern edge of the Edwards Plateau from multiple refugia along the Balcones Escarpment. There was little evidence for population and geographical expansion. Species distribution models predicted substantial reductions in suitable epigean habitat for *P. reddelli* at the Last Glacial Maximum (LGM).

**Conclusions:**

High genetic diversity, strong phylogeographical structure, diffusion from multiple refugia, and unfavourable climatic conditions at the LGM collectively support the hypothesis that caves served as microrefugia for *P. reddelli*. Similar patterns of genetic structure in *P. reddelli* and other troglophilic species across the Edwards Plateau karst region of Texas suggest that caves serving as microrefugia are important for the formation, maintenance, and future survival of troglophilic species in temperate karst regions.

## Background

The dramatic climatic oscillations of the Pleistocene profoundly impacted the geographical distribution and genetic structure of species in North America [1-3]. Parts of northern North America were periodically covered with glacial ice during the Pleistocene, resulting in dramatically fluctuating biotic communities [4]. As a result of distributional shifts, fragmentation of primary habitats, and the development of unfavourable climate conditions that exceeded physiological tolerances, many temperate species responded to Pleistocene climatic fluctuations with latitudinal shifts into and out of refugia [5]. Characterizing the location and extent of these climatic refugia has been the focus of phylogeographical research for decades (e.g. [1,6-8]). Climatic refugia are generally considered to be large regions in which species took refuge during glacial advances [3]. In North America such regions exist in the southeastern United States and in the Chihuahuan and Sonoran Deserts [8,9].

Evidence suggests, however, that climatic refugia also occurred at local scales [10]. Microrefugia are small areas “with local favourable environmental features, in which small populations can survive outside their main distribution area, protected from the unfavourable regional environmental conditions” ([11]: pp. 482–483). The climatic conditions of microrefugia differ from those of the surrounding region in a manner conducive to the persistence of species that may not survive elsewhere [10,12]. The survival of species within microrefugia has broad implications for how species responses to past climate change are viewed. Rather than invoking unrealistic long-distance dispersal (e.g. [13]) or ecological niche shifts (e.g. [14]) to explain discordant genetic structure and predicted paleo-distributions, species may simply have persisted in scattered microrefugia that supported isolated, low-density populations beyond their inferred distributional boundaries during the Pleistocene [12].

An important characteristic of microrefugia is a microclimate that is both stable and sufficiently distinct from the surrounding habitat [10,12]. Subterranean environments such as caves provide conditions that enable species to persist during fluctuating climate [15-17]. The long-term persistence of troglobionts (obligate cave dwellers; [18]) in regions impacted by Pleistocene glaciation has been documented in several European (e.g. [19]) and North American (e.g. [20-22]) taxa. Presumably, troglophiles, uniquely able to exploit both cave and surface habitats [18], could also utilize caves as microrefugia during unfavourable climate [23]. However, the concept that caves are potential microrefugia has not been well explored, perhaps owing to the relatively few phylogeographical studies of troglophilic species in temperate regions (e.g. [24-28]).

**Figure 1:**
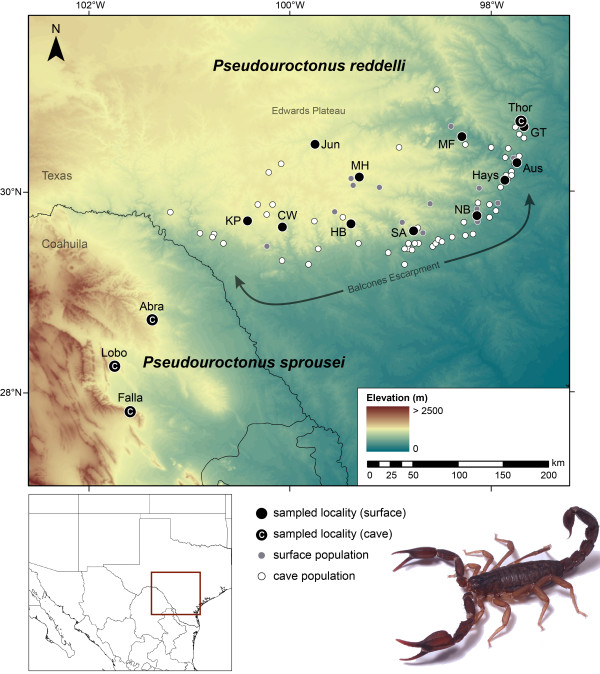
**Known localities of the North American vaejovid scorpions *Pseudouroctonus reddelli *and *Pseudouroctonus sprousei*.** Sampled localities indicated with black circles (abbreviations in Table 3). Other localities indicated with grey circles (surface populations) and white circles (cave populations), based on records from the literature [29,30,74,75]. The Balcones Escarpment that forms the southern edge of the Edwards Plateau in Texas is delineated.

The Texas cave scorpion, *Pseudouroctonus reddelli* (Gertsch & Soleglad 1972), of the endemic North American family Vaejovidae Thorell, 1876, occurs on humid rocky hillsides and in subterranean habitats throughout the karst of the Edwards Plateau of Texas [29] (Figure 1). Although the majority of known localities are caves, *P. reddelli* exhibits no apparent ecomorphological adaptations for life in caves [30,31] and can be common on the surface in rocky areas with oak leaf litter [29,30]. *Pseudouroctonus sprousei* Francke & Savary 2006, the sister species to *P. reddelli*[31], is troglobiotic and known from isolated caves in the arid Chihuahuan Desert in the Mexican state of Coahuila to the southwest of the Edwards Plateau.

We hypothesize that the ability of *P. reddelli* to exploit both epigean (surface) and hypogean (subterranean) habitats allowed this species to persist across their present distribution during Pleistocene climatic fluctuations. During favourable climatic conditions, the geographical distribution of *P. reddelli* across epigean habitats and genetic connectivity among populations would have been more extensive than during periods of unfavourable conditions, when epigean populations would have diminished while hypogean populations persisted in isolated microrefugia. Cyclical patterns of dispersal into and out of microrefugia during glacial–interglacial cycles, repeated throughout the Pleistocene, should produce a signature of marked genetic diversity and phylogeographical structure [12]. Genetic diversity is predicted to be retained in microrefugia during periods of unfavourable climate rather than lost, as during the extinction of recently-expanded populations with new genetic mutations [12]. Populations in scattered microrefugia represent remnants of the most recent population expansion and are predicted to be well-differentiated [12].

In this paper, we examine the phylogeography of *P. reddelli* using mitochondrial and nuclear DNA sequence data generated from 12 populations. We calculate descriptive measures of genetic diversity and population genetics statistics, and reconstruct phylogeographical structure, spatial distribution and population sizes through time using coalescent-based methods. We create species distribution models (SDMs) to infer the climatic niche of epigean populations of *P. reddelli* and their potential distribution both presently and during the Last Glacial Maximum (LGM). We test the hypothesis that caves served as microrefugia, which predicts marked genetic diversity and phylogeographical structure, spatial diffusion from multiple refugia during diversification, and SDMs portraying dramatically reduced or displaced suitable climatic conditions at the LGM. Other combinations of low genetic diversity, signatures of recent population expansion from large refugial areas, and SDMs depicting reduced suitable habitat, or of high genetic diversity, limited phylogeographical structure, a single refugial area, and SDMs portraying no reduction or displacement of suitable climatic conditions, would support alternative hypotheses. Finally, we compare phylogeographical patterns in *P. reddelli* with other troglophilic species across the Edwards Plateau karst region of Texas.

## Methods

### Taxon sampling and DNA sequencing

We generated DNA sequence data from 47 samples of *P. reddelli* collected from 12 localities across its range (Figure 1, Table 1). We obtained samples from 11 surface localities and one cave locality. *Pseudouroctonus reddelli* lacks apparent ecomorphological modifications for life in caves and most cave localities are generally near surface localities (Figure 1). In addition, gene flow between adjacent surface and cave populations of troglophiles is often high [23]. We therefore assumed that our sampling adequately captured range-wide genetic diversity. We also generated sequences from three *P. sprousei* samples from neighbouring Coahuila, Mexico.

**Table 1:** **Locality data for genetic samples of the North American vaejovid scorpions *Pseudouroctonus reddelli *** **and *Pseudouroctonus sprousei*, deposited in the Ambrose Monell Cryocollection at the American Museum of Natural History, New York**

**Sample ID**	***n***	**Species**	**Locality**	**Latitude**	**Longitude**
Austin	4	*P. reddelli*	U.S.A.: Texas: Travis Co.: Austin	30.2927	−97.7491
Camp Wood	4	*P. reddelli*	U.S.A.: Texas: Edwards Co.: W Camp Wood	29.6520	−100.0749
Georgetown	5	*P. reddelli*	U.S.A.: Texas: Williamson Co.: Georgetown	30.6480	−97.6848
Haby Cemetery	5	*P. reddelli*	U.S.A.: Texas: Bandera Co.: E Haby Cemetery	29.6832	−99.3868
Hays City	1	*P. reddelli*	U.S.A.: Texas: Hays Co.: Hays City	30.1246	−97.8686
Junction	4	*P. reddelli*	U.S.A.: Texas: Kimble Co.: Junction	30.4768	−99.7576
Kickapoo	2	*P. reddelli*	U.S.A.: Texas: Edwards Co.: NE Kickapoo Caverns	29.7166	−100.4275
Marble Falls	4	*P. reddelli*	U.S.A.: Texas: Burnet Co.: SW Marble Falls	30.5490	−98.2920
Mountain Home	5	*P. reddelli*	U.S.A.: Texas: Kerr Co.: SE Mountain Home	30.1487	−99.3165
New Braunfels	5	*P. reddelli*	U.S.A.: Texas: Comal Co.: N New Braunfels	29.7656	−98.1412
San Antonio	6	*P. reddelli*	U.S.A.: Texas: Bexar Co.: NW San Antonio	29.6075	−98.7791
Thor’s Cave	1	*P. reddelli*	U.S.A.: Texas: Williamson Co.: NW Georgetown	30.6826	−97.7077
Cueva Falla	1	*P. sprousei*	Mexico: Coahuila: Cueva de la Falla	27.8134	−101.5902
Cueva Lobo	1	*P. sprousei*	Mexico: Coahuila: Cueva del Lobo	28.2667	−101.7543
El Abra	1	*P. sprousei*	Mexico: Coahuila: Cueva El Abra	28.7334	−101.3791

We extracted genomic DNA from leg muscle tissue and generated sequences using standard protocols [32,33]. We sequenced two markers, including fragments of the mitochondrial (mtDNA) genes Cytochrome *c* Oxidase subunit I (*COI*; 754 base pairs, bp) and 16S ribosomal DNA (*16S*; 398 bp), and a contiguous region of nuclear DNA we collectively referred to as ITS that included partial 18S rDNA (123 bp), internal transcribed spacer 1 (403 bp), 5.8S rDNA (142 bp), internal transcribed spacer 2 (209–211 bp, 156 bp used in the final alignment, see below), and partial 28S rDNA (17 bp). We edited and manually aligned sequences for each individual using Sequencher v5.0 (Gene Codes Corporation, Ann Arbor, MI). We used PHASE v2.1.1 [34] to phase heterozygous sites within ITS, and retained the most probable pair of alleles for each heterozygous individual. We used three independent recombination tests (RDP, GENECOV, and MaxChi) in the program RDP v3.44 [35] to test for recombination within ITS. We applied default settings for all three methods.

### Genetic diversity

We calculated descriptive measures of genetic diversity for each locus, including the number of unique haplotypes (*h*), haplotype diversity (*H*_*d*_), and nucleotide diversity (π), in DnaSP v5.1 [36]. We calculated several classical population genetics statistics to further test for departures from equilibrium conditions. We calculated Tajima’s *D*[37] and Fu’s *F*_*s*_[38] using DnaSP. Negative and significant values reject the null hypothesis of population stasis. We calculated the sum of squares deviation (SSD) and Harpending’s raggedness index (RI) for the observed mismatch distributions using ARLEQUIN v3.5.1.3 [39], and compared with data simulated under a sudden expansion model. Significant *P*-values indicate rejection of the recent expansion hypothesis. We derived calculations from each complete dataset and separately for localities with sample sizes greater than or equal to four. We treated samples from Thor’s Cave (*n* = 1) and Marble Falls (*n* = 5), which are 4.4 km apart, as a single population, and excluded Kickapoo (*n* = 2) and Hays City (*n* = 1) from the analyses.

We used a series of linear regressions to test for possible geographical gradients in genetic diversity. We calculated the Pearson’s correlation coefficient (*r*) in R v2.15.2 [40] to test for correlations between diversity indices (*H*_*d*_ and π) and latitude, longitude, and sample size for individual localities. We tested significance with 1000 permutations.

### Phylogeographical structure

We estimated phylogeographical structuring and divergence dates independently for each locus using a relaxed Bayesian molecular clock framework implemented in BEAST v1.7.4 [41]. We used jModeltest v0.1.1 [42] to select the best-fit model of evolution, based on Akaike Information Criteria (AIC), for each of the two mtDNA genes. We calibrated the mtDNA tree by setting the clock-rate prior for each gene to a uniform distribution with bounds of 0.00734 and 0.00393 (*COI*) and 0.00486 and 0.00260 (*16S*). These values represent the number of substitutions per site per million years, derived from the inferred split of the vaejovid sister species *Konetontli pattersoni* (Williams 1980) and *Konetontli nayarit* (Armas & Martín-Frias 2001), presumably caused by geological separation of the Cape region of the Baja California Peninsula from mainland Mexico [32]. We calculated maximum likelihood-corrected pairwise divergences between *K. pattersoni* and *K. nayarit* for *COI* and *16S* sequences (GenBank accession numbers JX909605, JX909604, JX909531, and JX909530) using MEGA v5.1 [43], and divided the resultant numbers by 14 and 7.5 Ma, the maximum and minimum ages proposed for the separation of the Cape from mainland Mexico [44,45]. We specified a strict clock for each gene after verification of clock-like rates using a stepping-stone model and a Bayes factor comparison in MrBayes v3.2.1 [46]. We used jModeltest to estimate the best-fit model of evolution for the ITS data. We did not partition the ITS gene region to avoid over-parameterization, given the relatively low total number of informative sites. We derived the substitution rate for ITS as above using *K. pattersoni* and *K. nayarit* and *ITS2* sequences (GenBank accession numbers JX909406 and JX909405) from Bryson *et al.*[32]. We applied a uniform distribution with bounds of 0.00424 and 0.00187 on the clock-rate prior. We conducted analyses for 40 million generations, with samples retained every 1000 generations, using constant size coalescent tree priors for the mtDNA and the ITS loci. We displayed results in TRACER v1.5 [47] to confirm acceptable mixing and likelihood stationarity, appropriate burn-in, and adequate effective sample sizes (ESS) above 200 [48] for all estimated parameters. We summarized the parameter values of the samples from the posterior distribution on the maximum clade credibility tree, after discarding the first 4 million generations (10%) as burn-in, using TreeAnnotator v1.7.4 [41].

Our divergence dates are based on estimates from a single calibration point (the inferred split of *K. pattersoni* and *K. nayarit*). We therefore conducted additional analyses with the mtDNA data using the same priors and scorpion-specific mutation rates of 0.005 substitutions/site/million years for *16S*[49] and 0.007 substitutions/site/million years for *COI*[50]. Although this ‘scorpion clock’ was calculated from buthid scorpions distantly related to *P. reddelli*, it has been used to estimate divergences within other scorpions [33,51,52], and no other mutation rates have been estimated for scorpions to date. We conducted analyses for 40 million generations and retained samples every 1000 generations.

We also assessed spatial genetic structure by testing the significance of isolation by distance (IBD) across the distribution of *P. reddelli* using the matrices of pairwise population differentiation statistics (*F*_*ST*_) and the natural logarithm of the Euclidian geographical distances. We calculated *F*_*ST*_ values for genetic distances with ARLEQUIN for each locus, and generated Euclidian distances with the IBRWS web server 0.1 [53]. We subsequently used the IBD web server v3.23 [54] to check for isolation-by-distance by performing a Mantel test with 1000 random permutations.

### Phylogeography through time

We inferred the geographical origin of *P. reddelli* and its spatial distribution during diversification using a relaxed random walk (RRW) diffusion model across continuous space in BEAST. This model incorporates uncertainty in the tree topology and the spatial diffusion process to reconstruct time-sliced contours from the posterior tree distribution that represent the credibility intervals for locations at any point in time [55]. We included the sister species *P. sprousei* and ran independent analyses on each locus. We edited the xml file, prior to analyses, to input random noise to offset identical coordinates using the jitter option with a parameter of 0.01. We used the gamma RRW model for the continuous trait model prior. We performed analyses on the mtDNA dataset for 40 million generations using the BEAST priors specified above. We performed two independent analyses on the ITS dataset due to low ESS values (see below), each for 400 million generations with samples retained every 10,000 generations. We then combined posterior parameter estimates across the two runs using LogCombiner v1.7.4 [41]. We estimated a maximum clade credibility tree using TreeAnnotator and projected it onto the grid of geographical coordinates using SPREAD v1.0.5 [56] to visualize phylogeographical reconstructions for each locus. We visualized the kml file output by SPREAD in Google Earth v6.0.1 (Google Inc.).

### Historical demography through time

We generated coalescent-based Gaussian Markov random field (GMRF) Bayesian skyride plots [57] with BEAST to estimate the magnitude and relative timing of changes in effective population size since the time of the most recent common ancestor. This method differs from the similar Bayesian skyline plot [58] in that the former does not require specification of strong user-defined priors for the number of population size changes during the history of the sample [57]. We performed independent analyses on the mtDNA and ITS datasets, without *P. sprousei*, using time-aware smoothing. Priors for the mtDNA and ITS datasets were as specified previously, but we used jModeltest to produce new best-fit models of evolution estimated for the ingroup only. We ran each dataset for 40 million generations, with samples retained every 1000 generations. We checked output files in TRACER to verify proper mixing and ESS values greater than 200 for each parameter. We subsequently reconstructed GMRF skyride plots in TRACER. Because strong population structure can violate skyride model assumptions of panmixia and potentially bias reconstructions [59], we conducted additional analyses on the two strongly supported subclades of *P. reddelli* (see below) using the mtDNA data. Regardless, we interpreted results cautiously and used them to cross-validate our descriptive statistics of departures from population size constancy (Tajima’s *D,* Fu’s *F*_*s*_, sum of squares deviation, and Harpending’s raggedness index).

### Species distribution modelling

#### Climate data

We obtained data for the climate of the study area from the WorldClim website (http://www.worldclim.org), at a resolution of 30 arc sec, approximately 1 km. We chose this resolution because of the sub-continental extent of the study, the relatively restricted distribution of the species within the study area, and the availability of high-resolution geographical coordinate data for species occurrence records. These climate data for North America represent an average over the period of approximately 1960–1990 [60]. We refer to this as present climate. Although estimates of local epigean climate could have provided information on climatic conditions directly experienced by *P. reddelli*, such data were unavailable for present climate or for climate modelled for the LGM. We used the results of two ocean–atmosphere coupled general circulation models (GCMs) as estimates for climate at the LGM, MIROC (A Model for Interdisciplinary Research On Climate) and CCSM (Community Climate System Model). The modelled results were produced as part of the PMIP2 model comparison project [61] and downscaled versions were available at 2.5 arc min resolution from the WorldClim website. We further downscaled these raster layers using an inverse distance weighting algorithm in ArcMap 10 (ESRI, Redlands, CA, USA) to interpolate the LGM climate data to 30 arc sec [62]. We represented present and LGM climate with 19 ‘bioclimatic’ variables (see http://www.worldclim.org), which may bear a closer relationship with physiological limitations than simple monthly means [60].

#### Ensemble approach and algorithms

We chose an ensemble approach to distribution modelling in order to account for potential model-based uncertainty [63,64]. We chose four commonly used modelling algorithms. Two of these, generalized linear models (GLM) [65] and generalized additive models (GAM) [66], represented inherently statistical approaches whereas the others, boosted regression trees (BRT) [67] and maximum entropy (MaxEnt) [68,69], were machine-learning algorithms. Generalized linear models and GAMs were implemented in R, using the packages *stats* and *gam*[70,71], and BRTs and MaxEnt using the packages *gbm* and *dismo*, respectively [72,73].

We used 25 occurrences of *P. reddelli* obtained from literature records [30,74,75] and samples collected during the study (Table 1) for developing distribution models. Each point was a surface locality geo-referenced to within several tens of metres, and no two points occupied the same pixel of the 1 km-resolution climate data. These occurrences constituted presence-only data and as such it was necessary to identify an encompassing area for the selection of climate background (MaxEnt) and pseudo-absence data (other algorithms). Although the role of these data differs among algorithms, we refer to them generically as pseudo-absence data. We chose an areal extent in southern North America (primarily the southern United States and Mexico) where we considered it reasonable that suitable climatic conditions for *P. reddelli* might be found presently and during the LGM. This was important because the inclusion of excessively long environmental gradients in pseudo-absence selection (e.g. by choosing the entirety of North America) may substantially over-predict the extent of distributions when modelling with presence-only data [76]. We randomly chose 2000 locations from the selected area using a function in R to provide pseudo-absence data. The use of several thousand randomly selected pseudo-absence points generally leads to high model performance across algorithms [77]. These data were weighted such that species prevalence in BRTs, GLMs and GAMs was effectively 0.5. Manual weighting is not possible in MaxEnt, so we selected the default prevalence value of 0.5. All cropping, downscaling and extraction of gridded climate values was done with the R packages *raster*[78], *sp*[79], and *Rgdal*[80].

#### Overfitting, parameterization and multi-colinearity

We constrained the models in several ways to avoid overfitting the current distribution of *P. reddelli* to the environmental data. This was important because close conformance to the training data by overfitted models (i.e. fitting error variation) may cause models to perform poorly when projected to other areas or climate datasets. The algorithm in the *gbm* package produces models by sequentially fitting simple regression and decision trees to the occurrence and pseudo-absence data. We ensured the use of simple trees by limiting tree depth to three. We used a conservative fitting criterion (‘oob’) [81] and adjusted the tree weightings (‘learning rate’ in the *gbm* package) to reduce the chance of overfitting and ensure that the number of trees in each final BRT model was between 2000 and 8000. We set the degrees of freedom in GAMs to three. We also set the synthetic variables generated by MaxEnt (‘features’) to a single type (‘hinge’). We included quadratic terms for each variable in GLM models, but did not include terms expressing interactions among variables, which may also prevent obtaining models that closely fit particular correlation structures among climate variables.

The projection of SDMs to climates that differ from those used in model calibration may be complicated when climate data differ strongly in the correlation structure among the variables used in distribution models. We mitigated the tendency of algorithms to fit a particular correlation structure in climate data by limiting the number of climate variables to four when parameterizing each model. We also limited the degree of multicolinearity among the chosen variables by ensuring that the maximum variance inflation factors of the sets of four variables were less than 4.0 and the pairwise correlations of r_p_ were less than 0.7. We fitted 20 models as the factorial combination of the four algorithms with five sets of uncorrelated climate variables. Each set contained two precipitation and two temperature variables (Table 2). We evaluated model performance with statistics commonly used for judging the performance of SDMs: the Area Under the Curve of the Receiver Operator Characteristic Function (AUC) [82], the proportion of presences and absences correctly categorized (pcc), the proportion of presences correctly predicted (sensitivity), and the proportion of absences correctly predicted (specificity). Each statistic was estimated for each model using 10-fold cross-validation on randomly chosen sub-samples of the training data (maintaining original species prevalence) and then averaging the values.

**Table 2:** **Climate variables from the WorldClim website (http://www.worldclim.org) used for parameterizing each of 20 distribution models for the North American vaejovid scorpion *Pseudouroctonus reddelli***

		
(a)	Sets	WorldClim variables
	1	Bio3, Bio8, Bio15, Bio18
	2	Bio1, Bio2, Bio8, Bio17
	3	Bio2, Bio5, Bio18, Bio19
	4	Bio5, Bio6, Bio13, Bio15
	5	Bio2, Bio8, Bio11, Bio14
(b)	Variable name	Variable definition
	Bio1	Annual mean temperature
	Bio2	Mean diurnal temperature range
	Bio3	Isothermality
	Bio5	Maximum temperature of the warmest month
	Bio6	Minimum temperature of the coldest month
	Bio8	Mean temperature of the wettest quarter
	Bio11	Mean temperature of the coldest quarter
	Bio13	Precipitation of the wettest month
	Bio14	Precipitation of the driest month
	Bio15	Precipitation seasonality
	Bio17	Precipitation of the driest quarter
	Bio18	Precipitation of the warmest quarter
	Bio19	Precipitation of the coldest quarter

(a) Variable sets used in modelling and (b) variable definitions.

Predictions of the current distribution of the species were formulated by determining probabilities of species presence (we used habitat suitability values for MaxEnt), conditioned on the current climate values for each 1 km pixel. We converted these continuous values to a Bernoulli variable that represented the presence or absence of suitable climate by tabulating model probabilities against the presence and pseudo-absence values used in model calibration. We subsequently determined the threshold probability as the value that maximized the True Skill Statistic (TSS), which previously demonstrated performance independent of species prevalence and has often been used for this purpose [83]. The use of a flexible statistic to determine the probability threshold for predicted presence has proven superior to the use of a fixed, arbitrary threshold [84]. We used the corresponding TSS value for each SDM to determine the predicted distribution of suitable climate at the LGM. We emphasized the degree of consensus among species distribution models regarding prediction of suitable climate for the species by overlaying the mapped predictions of each model to create a spatial frequency distribution.

## Results

### Genetic data

Parsimony-informative sites comprised 13.5% of the mtDNA dataset (*COI:* 111/754 bp; *16S*: 45/398 bp). The nuclear ITS gene region contained less variation than the mtDNA: 10% of the sites were parsimony-informative (84/841 bp). We were unable to obtain ITS sequences from the single sample from Hays City and one sample each from Junction, Kickapoo, and Camp Wood. Nuclear data were also missing for *P. sprousei* from El Abra. More than half of the *P. reddelli* samples (82%) were heterozygous for either one or two single base-pair indels within a 55-bp segment near the beginning of the *ITS2* gene. Chromatograms on either side of this region (in the forward and reverse primers, respectively) were easy to read and align across all samples. We therefore discarded the intervening 55-bp section from the dataset. None of the three methods used in RDP detected recombination in the ITS dataset. We deposited sequence data in GenBank (KF982915–KF983014) and sequence alignments in the Dryad Digital Repository (http://dx.doi.org/10.5061/dryad.66rd8).

### Genetic diversity

Thirty-two unique mtDNA haplotypes were found among the 47 samples of *P. reddelli* sequenced, none of which were shared among localities. Thirty-two of the 86 phased ITS alleles were unique and twelve were not shared among localities (Austin, 4; San Antonio, 3; New Braunfels, 2; and Junction, Mountain Home, and Camp Wood, 1 each). Mean haplotype and nucleotide diversity estimates were relatively high: 0.9800 (*H*_*d*_) and 0.0156 (π) for all mtDNA samples combined; 0.8860 (*H*_*d*_) and 0.0042 (π) for ITS. The *H*_*d*_ and π estimates for sample localities indicated the lowest genetic diversity values were observed in the Marble Falls and Junction populations (Table 3). There were no significant correlations between diversity indices and longitude or between diversity indices and sample size (Additional file 1). Similarly, no significant correlations were found between π and latitude. However, there were significant correlations between *H*_*d*_ and latitude in both loci (mtDNA: *r* = −0.6718, *P* = 0.0475; ITS: *r* = −0.7603, *P* = 0.0174).

**Table 3:** **Measures of genetic diversity for the North American vaejovid scorpion *Pseudouroctonus reddelli*, including sample size (*n*), number of unique haplotypes (*h*), haplotype diversity (*H***_***d***_**), nucleotide diversity (π), Tajima’s** ***D*, Fu’s *F***_***s***_**, sum of squares deviation (SSD), and Harpending’s raggedness index (RI)**

	**mtDNA**								**ITS**							
**Locality**	***n***	***h***	***H***_***d***_	**π**	***D***	***F***_***s***_	**SSD**	**RI**	***n***	***h***	***H***_***d***_	**π**	***D***	***F***_***s***_	**SSD**	**RI**
GT	6	3	0.6000	0.0051	−1.3152	3.3578	0.2035	0.4533	12	5	0.7880	0.0032	2.2171	0.4605	0.0336	0.0826
Aus	5	4	0.9000	0.0093	−0.2287	1.7144	**0.2151**	0.5900	10	6	0.8440	0.0047	−0.2812	−0.1484	0.0468	0.1269
MF	4	2	0.5000	0.0004	−0.6124	0.1719	0.0219	0.2500	8	3	0.7140	0.0012	1.1039	0.2043	0.0087	0.1225
NB	5	4	0.9000	0.0056	0.8036	0.8832	**0.2112**	0.5900	10	7	0.9330	0.0050	−0.0445	−1.1634	0.0491	0.1437
SA	6	4	0.8670	0.0064	0.2878	1.9308	0.1831	**0.4889**	12	8	0.9390	0.0039	0.4190	−2.2709	0.0611	0.1607
HC	5	5	1.0000	0.0043	−0.9543	−1.3451	0.0609	0.1200	10	4	0.8220	0.0034	1.4113	1.4153	0.0088	0.0385
MH	5		0.8000	0.0097	−0.4962	4.0693	0.2350	0.5200	10	4	0.7330	0.0024	0.5276	0.5924	0.0711	0.2064
Jun	4	1	0.0000	0.0000	0.0000	0.0000	0.0000	0.0000	6	3	0.7330	0.0047	1.6471	2.3987	0.1648	0.2756
CW	4	3	0.8330	0.0035	0.4671	1.1632	0.2223	0.7500	6	4	0.8670	0.0020	1.3861	−0.8336	0.0758	0.2933
All	47	32	0.9800	0.0156	−0.4569	−3.5808	0.0032	**0.0109**	86	32	0.8860	0.0042	−0.8142	**−20.6848**	0.0038	0.0120

Values of *F*_*s*_ and Tajima’s *D* in boldface indicate rejection of the hypothesis of constant population size (*P* < 0.05). SSD and RI values in boldface indicate failure to reject the hypothesis of recent expansion (*P* > 0.05). Sampled localities are abbreviated as follows: Georgetown (GT), Austin (Aus), Marble Falls (MF), New Braunfels (NB), San Antonio (SA), Haby Cemetery (HC), Mountain Home (MH), Junction (Jun), and Camp Wood (CW). Sample size for the nuclear internal transcribed spacer region (ITS) represents the number of phased alleles.

Descriptive statistics of departure from population size constancy were ambiguous, but no combinations of the four independent estimates strongly supported population expansion. Tajima’s *D* and Fu’s *Fs* values could not reject the null hypothesis of population stasis with one exception (Table 3). In this case, Fu’s *F*_*s*_ statistic was significantly negative for the ITS data combined (*F*_*s*_ = −20.6848, *P* < 0.01). The SSD and RI calculated for the observed mismatch distributions (Table 3) failed to reject the null hypothesis of recent population expansion for three populations based on mtDNA (SSD: Austin, New Braunfels; RI: San Antonio) and for the complete mtDNA dataset (RI).

### Phylogeographical structure

**Figure 2:**
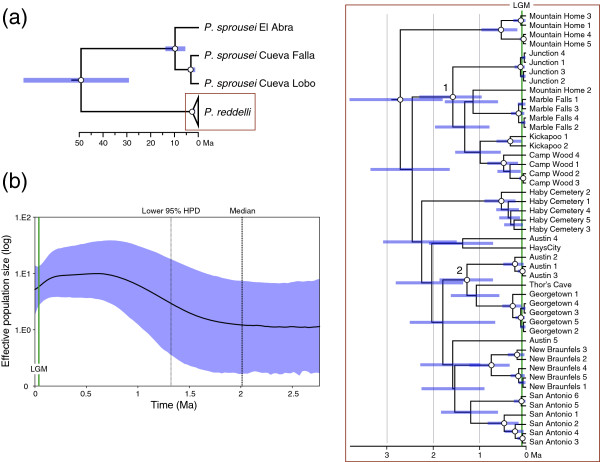
**Phylogeographical structure and demographic reconstruction through time inferred from 1,152 base pairs of mitochondrial DNA for the North American vaejovid scorpions *Pseudouroctonus reddelli *and *Pseudouroctonus sprousei *. (a)** Time-calibrated Bayesian gene tree for *P. reddelli* and *P. sprousei*. Nodes that received ≥ 0.95 posterior probability support depicted with white dots. Two strongly supported subclades of *P. reddelli* are numbered (1 and 2). **(b)** Bayesian skyride plot for *P. reddelli*, showing change in effective population size of females since the time of the most recent common ancestor. HPD = highest posterior density; LGM = Last Glacial Maximum.

The GTR + I + G (*CO1*) and HKY + I + G (*16S* and ITS) models of sequence evolution were selected using jModeltest. Initial analyses of the mtDNA dataset revealed low ESS values (< 100) for several parameters associated with the GTR model. We therefore used the less complex HKY model in final analyses, which resulted in higher (> 200) ESS values. The mtDNA gene tree (Figure 2a) indicated strong phylogeographical structure across the distribution of *P. reddelli*. Samples from most populations grouped together; only samples from Austin and Mountain Home were not monophyletic. Relationships among populations were generally unsupported throughout the tree. However, two strongly supported subclades were evident. One subclade contained the Junction, Marble Falls, Kickapoo, and Camp Wood samples along with one sample from Mountain Home. The second subclade was comprised of the Georgetown samples, the sample from nearby Thor’s Cave, and three samples from Austin.

Divergence dates estimated using the vaejovid calibration and the buthid ‘scorpion clock’ calibration differed (Table 4). Divergences inferred using the vaejovid calibration were older, and this was especially evident at the base of the tree. However, differences between mean estimated divergence dates within *P. reddelli* were relatively minor, ranging from 0.5 Ma (at the most recent common ancestor of *P. reddelli*) to 0.03 Ma (at the most recent common ancestor of the Marble Falls population), and all were within the Pleistocene (Table 4). We consider results based on the vaejovid calibration to be our best estimate of divergence dates because the species used to derive the calibration were vaejovid scorpions more closely related to *P. reddelli* and *P. sprousei*, but we acknowledge the drawbacks of basing conclusions on a single calibration derived from taxa outside of the focal group [85].

**Table 4:** **Estimated divergence dates based on two different calibration methods using 1,152 base pairs of mitochondrial DNA for the North American vaejovid scorpions *Pseudouroctonus reddelli *** **and *Pseudouroctonus sprousei***

**Node**	**Baja vaejovid calibration**	**buthid ‘scorpion clock’**
*P. sprousei*/*P. reddelli*	49.25 (28.94–73.24)	39.16 (24.85–54.95)
*P. reddelli*	2.58 (1.70–3.61)	2.08 (1.51–2.67)
Subclade 1	1.45 (0.86–2.13)	1.17 (0.76–1.62)
Subclade 2	1.40 (0.78–2.07)	1.13 (0.69–1.57)
New Braunfels	0.81 (0.37–1.31)	0.64 (0.31–0.99)
Marble Falls	0.16 (0.02–0.34)	0.13 (0.02–0.27)

Referenced nodes are indicated in Figure 2. Divergence times in millions of years are followed by 95% posterior density intervals in parentheses.

Divergence time estimates using our preferred vaejovid calibration contained wide 95% highest posterior density (HPD) intervals, especially at the base of the tree. The split between *P. sprousei* and *P. reddelli* occurred tens of millions of years before the Pleistocene (mean = 49.3 Ma; 95% HPD = 28.9–73.2 Ma). Early diversification within *P. reddelli* probably began near the Pliocene–Pleistocene boundary around 2.6 Ma. Subsequent divergences within *P. reddelli* based on mean estimates all occurred within the Pleistocene, but predated the LGM at around 0.02 Ma.

**Figure 3:**
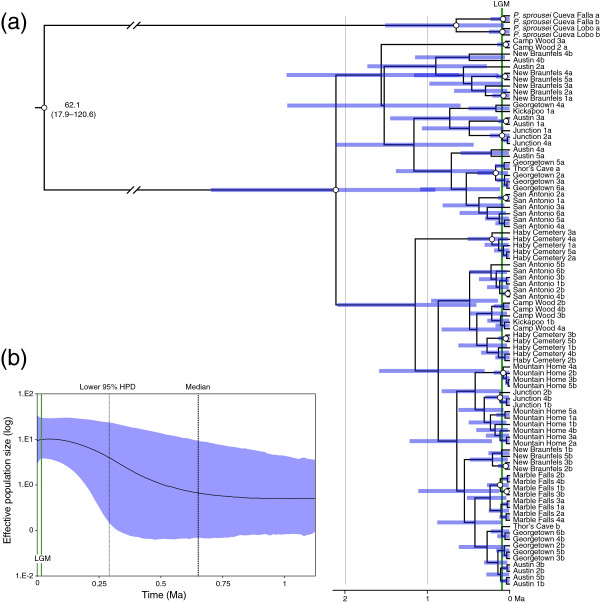
**Phylogeographical structure and demographic reconstruction through time inferred from 841 base pairs of the nuclear internal transcribed spacer region (ITS) for the North American vaejovid scorpions *Pseudouroctonus reddelli *and *Pseudouroctonus sprousei *. (a)** Time-calibrated Bayesian gene tree for *P. reddelli* and *P. sprousei*. Nodes that received ≥ 0.95 posterior probability support depicted with white dots. **(b)** Bayesian skyride plot for *P. reddelli*, showing change in effective population size since the time of the most recent common ancestor. HPD = highest posterior density; LGM = Last Glacial Maximum.

Strong phylogeographical structure within *P. reddelli* was also evident based on ITS data (Figure 3a). However, only one population (Marble Falls) was exclusively monophyletic and few nodes received greater than 0.95 posterior probability support. Mean estimates of divergence times were younger and 95% HPDs were wider than those based on the mtDNA data, but supported a relatively ancient divergence between *P. sprousei* and *P. reddelli*, a probable origin of *P. reddelli* near the beginning of the Pleistocene, and range-wide diversification within *P. reddelli* entirely within the Pleistocene, based on mean estimated divergence dates. Further, mean estimated divergences for all but one node predated the LGM. The limited resolution obtained is consistent with the relatively few phylogenetically informative characters and the large number of heterozygous positions within the ITS region (177 across 24 polymorphic sites). Visualization of the data in a network (Additional file 2) revealed no novel insights compared to the Bayesian tree-based approach. The Mantel test revealed no significant correlation between geographical and genetic distances in the mtDNA (*r* = 0.12, one-sided *P* = 0.26) and ITS (*r* = −0.29, one-sided *P* = 0.97) datasets. These results suggest that phylogeographical structure was probably not caused by differences in interpopulation gene flow across the range of *P. reddelli*.

### Phylogeography through time

**Figure 4:**
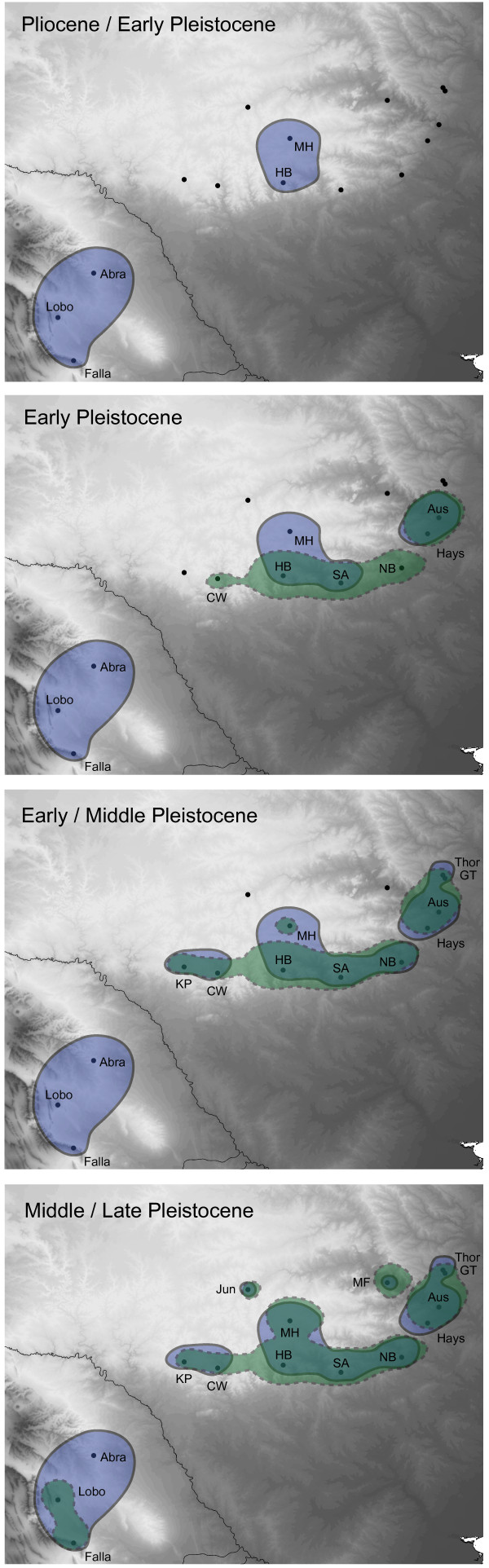
**Reconstructions of the spatial history of the North American vaejovid scorpions *Pseudouroctonus reddelli *and *Pseudouroctonus sprousei *based on Bayesian analyses of mitochondrial DNA (solid line) and nuclear internal transcribed spacer region (ITS) data (dashed line).** Polygons represent 80% highest posterior density intervals for the spatial location of ancestral populations across four time slices. Abbreviations for sampled localities in Table 3.

Preliminary BEAST analyses using the RRW model and the ITS data for 40 million generations, sampling every 1000 generations, resulted in low ESS values and poor convergence between analyses for three parameters associated with the RRW model (traitLikelihood, col1, and col2). We obtained higher ESS values (> 150) by performing analyses from different starting seeds for 400 million generations and sampling every 10,000 generations. We then combined posterior parameter estimates from two of these analyses that demonstrated convergence for all parameters using LogAnnotator. Visualization of the results from all independent analyses and the final combined analysis in GoogleEarth indicated similar phylogeographical patterns, differing only slightly in the extent of spatial uncertainty. The spatial distribution of *P. reddelli* from the final combined analysis corresponded reasonably well with spatial patterns from the mtDNA (Figure 4), further supporting the robustness of the data.

The spatial diffusion of *P. reddelli* through time, reconstructed from the mtDNA and ITS data (Figure 4) were in general similar, and differed mainly in the timing and extent of the ancestral origin of *P. reddelli*. Differences in timing were consistent with younger dates inferred from the ITS data in BEAST analyses. The mtDNA data suggested that *P. reddelli* originated near the centrally located Mountain Home and Haby Cemetery localities during the Early Pleistocene, about 2.5 Ma. Subsequent diffusion occurred along the southern edge of the Edwards Plateau from multiple refugia along the Balcones Escarpment. Final dispersal events to the northern Marble Falls and Junction localities occurred during the Late Pleistocene, around 0.1 Ma. Based on the ITS data, *P. reddelli* originated along the southern edge of the Edwards Plateau around 1.5 Ma. As with reconstructions based on the mtDNA data, final dispersal events occurred to the Marble Falls and Junction localities. Missing ITS data for the divergent sample of *P. sprousei* from El Abra may explain why *P. sprousei* appeared to originate late in the Pleistocene, rather than millions of years earlier as suggested by the mtDNA data.

### Historical demography through time

The GTR + I (*CO1*), HKY + G (*16S*), and HKY + I (ITS) models of sequence evolution were selected for the ingroup using jModeltest. However, as with the BEAST analyses discussed above, we changed the GTR model to the less complex HKY model for the *COI* partition to obtain adequate ESS values. Bayesian skyride plots reconstructed from the mtDNA (Figure 2b) and ITS (Figure 3b) data yielded similar results. The overall pattern was similar but, as with previous analyses, the estimated time was different; divergence date estimates based on ITS were younger than those based on mtDNA. Bayesian skyride plots for both loci suggested a relatively stable population size after origination, a gradual increase in population size (2–1 Ma during the Early Pleistocene, based on mtDNA, and 0.3–0.1 Ma during the Middle Pleistocene, based on ITS), and a return to relative population stasis (from the Late Pleistocene through the present). The mtDNA suggested a possible decrease in population size towards the end of the Pleistocene, but this may be an artefact of violating the model assumption of population admixture. This interpretation is further supported by the Bayesian skyride plots for the two mtDNA subclades (Additional file 3), both of which suggested relatively stable populations during this time. Bayesian skyride plots did not provide strong evidence for a dramatic population expansion, consistent with results of our other demographic analyses.

### Species distribution modelling

**Figure 5:**
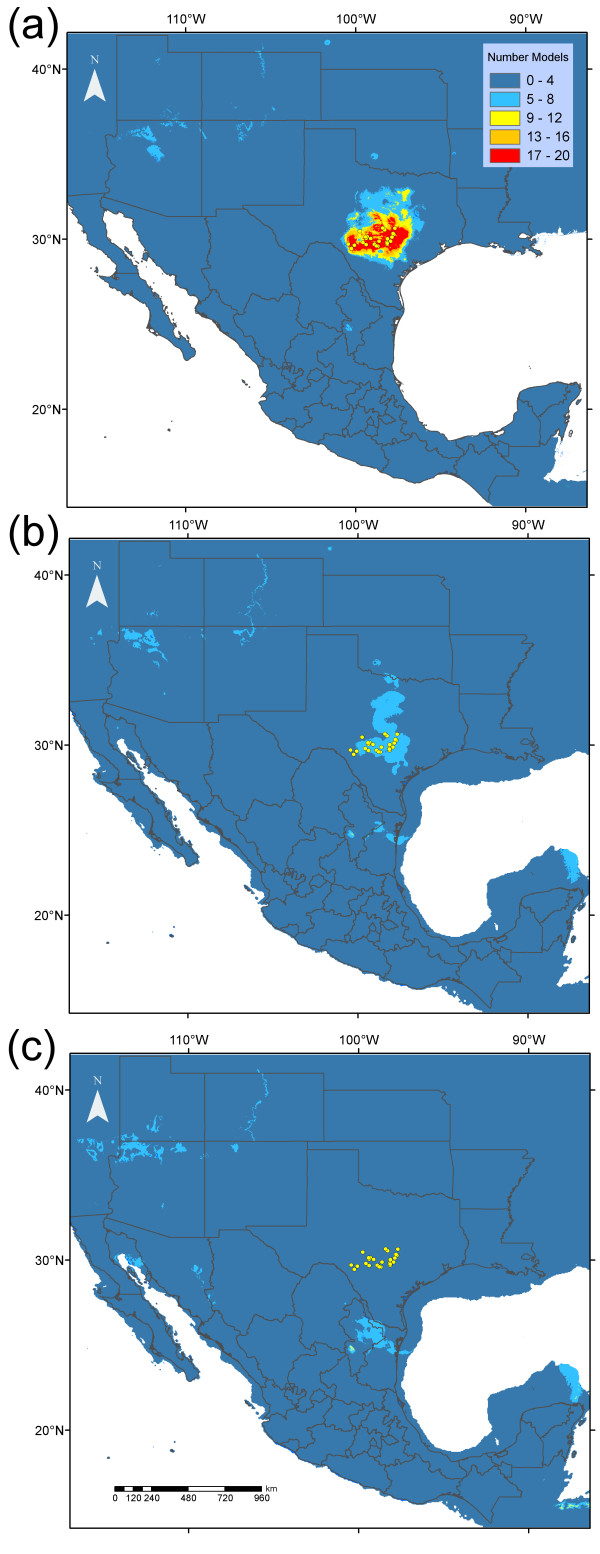
**Predicted distribution of suitable climate for the North American vaejovid scorpion *Pseudouroctonus reddelli*, produced by an ensemble of 20 species distribution models.** Models were the product of the factorial combination of four modelling algorithms with five sets of uncorrelated variables. Dots indicate the locations of 25 surface occurrences for *P. reddelli* used to develop distribution models. Colours indicate the number of models that predict the presence of suitable climate at present, as represented in gridded layers from WorldClim **(a)**, and during the Last Glacial Maximum, as represented by the CCSM **(b)** and MIROC **(c)** general circulation models.

The performance of the 20 models was high in 10-fold cross-validation of AUC (0.959 ± 0.010, mean ± sd). Determination of the threshold probability for predicted presence using TSS resulted in a mean proportion of correctly classified training observations of 0.925 ± 0.016. We observed that the sensitivity of models was in all cases maximal (1.0 ± 0.0), and values of specificity were also high (0.924 ± 0.016). A strong consensus of 17 to 20 models predicted a current distribution of suitable climate for *P. reddelli* that clustered tightly around the known locations used in model training (Figure 5a).

Consensus predictions of the distribution of suitable climate for *P. reddelli* during the LGM differed substantially from predictions of the distribution of presently suitable climate. Only 5 to 8 of the 20 models projected upon the LGM climate estimate produced by the CCSM GCM predicted the presence of suitable conditions across the present distribution of *P. reddelli* (Figure 5b). None of the 20 models projected upon the LGM climate estimate of the MIROC GCM predicted the existence of suitable climate across the present distribution of the species. Instead, 5 to 8 models predicted the existence of suitable conditions during the LGM approximately 400 km to the south of the present distribution (Figure 5c). The majority of models did not support the presence of suitable climate during the LGM in any part of the study area under either climate estimate.

## Discussion

### Phylogeography of *P. reddelli*

Results suggest a history of Pleistocene diversification within *P. reddelli* and support the hypothesis that caves served as microrefugia for this species. Multiple lines of genetic evidence support a general model of distributional stasis in *P. reddelli* over an alternative hypothesis of geographical expansion. Haplotype and nucleotide diversity are high (Table 3), substantial phylogeographical structure is evident (Figures 2 and 3), and the results broadly suggest population and geographical expansion was minimal (Table 3, Figures 2b, 3b, 4). High haplotype diversity in conjunction with high nucleotide diversity usually indicates a historically stable population [1,5] and is a genetic signature predicted by the microrefugia model [12]. Conversely, an ensemble of SDMs suggests that suitable epigean habitat for *P. reddelli* during the Pleistocene significantly decreased by the LGM and may have shifted several hundred kilometres to the south (Figure 5). The ability of *P. reddelli* to inhabit both surface and subterranean habitats probably allowed this scorpion to persist in caves scattered across its current distribution during cycles of unfavourable Pleistocene climate. Cave populations have likely retained genetic diversity, which has accumulated through time rather than been lost during changes in distribution. Such a scenario would explain the seemingly contradictory results from the genetic data and distribution models.

*Pseudouroctonus reddelli* probably diverged from its sister species, *P. sprousei*, during the Miocene and subsequently colonized the Edwards Plateau region in the early Pleistocene (Figures 2, 3 and 4). Similar deep divergences in the Miocene have been inferred for other North American vaejovid scorpions [32,33,52]. This colonization may have followed the Miocene–Pliocene formation of the Balcones fault zone and erosion of the Edwards Plateau into a karst region [86-88]. Results from the RRW diffusion models suggest populations then expanded out from multiple refugia along the cave-rich area of the Balcones Escarpment and reached current distributional limits by the late Pleistocene (Figure 4). Given the dramatic reduction in predicted suitable habitat at the LGM during the late Pleistocene (Figure 5), dispersal of *P. reddelli* from cave microrefugia may have occurred during warmer interglacial periods, but was arrested during cooler glacial periods, which accounted for about 80% of the Pleistocene [89]. Prolonged duration in cave microrefugia may also explain why most sampled localities showed no evidence of admixture and formed monophyletic groups based on the faster-sorting mtDNA (Figure 2). The last inferred dispersals were to the northern Marble Falls and Junction localities late in the diversification history of *P. reddelli*. This slight northward expansion would explain the significant correlation between haplotype diversity (*H*_*d*_) and latitude and the low genetic diversity values within the populations at both localities.

The observed high genetic diversity, strong phylogeographical structure, spatial diffusion from multiple refugia, and unfavourable climatic conditions at the LGM collectively support the hypothesis that caves served as microrefugia for *P. reddelli*. However, there may be alternative explanations for the pattern observed. Scorpions are generally poor dispersers which increases their propensity to show strong phylogeographical structure [18]. Although several recent studies of North American scorpions suggest that Pleistocene glacial–interglacial periods may have had relatively little impact on distributions and genetic structure [32,33], other studies have found strong evidence for recent postglacial range expansions and relatively weak phylogeographical structure [51,90-92]. These findings suggest that Pleistocene climate change had a potentially strong impact on the diversification of some North American scorpion species and that not all species exhibit strong phylogeographical structure. It is possible that *P. reddelli* persisted in scattered surface localities across the Edwards Plateau during Pleistocene glacial–interglacial periods, thus making caves insignificant for the survival of this species. A small minority of the total number of SDM models in fact predict fragmented patches of suitable habitat across the Edwards Plateau at the LGM (Figure 5). However, not every single model is expected to show the same pattern; this is the point of using an ensemble modelling approach. Climatic variation during stadial–interstadial cycling may also have affected the distribution of suitable climate, but no suitable climate models are available to examine this possibility. The evidence is strongly against the presence of suitable surface conditions during the LGM.

### Caves as microrefugia

The locations of microrefugia have important implications for phylogeographical research as they may influence perceptions about the genetic diversity and spatial distribution of species in response to climate change. Several abiotic factors impact the formation of microrefugia including slope, exposure, and elevation [10]. As a result, microrefugia in temperate regions often occur in regions of topographical complexity [10,12,93]. However, even relatively tiny areas with stable environments, such as aquatic springs, can serve as microrefugia [12]. Karst landscapes are pocketed with caves containing stable environments. Across temperate regions of the world, caves probably served as microrefugia for troglophilic species during the Pleistocene, as evidenced by the distribution of *P. reddelli* across the Edwards Plateau. During favourable environmental conditions, gene flow would have increased across the epigean habitats of troglophilic species [23]. As environmental conditions deteriorated and epigean populations diminished, cave populations would have persisted in isolated microrefugia. A cyclical pattern of dispersal into and out of cave microrefugia, in response to Pleistocene glacial–interglacial cycles, should produce marked genetic diversity and phylogeographical structure in troglophilic species. In addition, species distribution models should predict a dramatic reduction or shift in suitable habitat concurrent with climate oscillations associated with glacial–interglacial cycles. These expectations (high genetic diversity, strong phylogeographical structure, diffusion from multiple refugia, and significantly reduced or shifted Pleistocene surface habitats) should form the basis for future tests of the hypothesis that caves served as microrefugia for troglophilic species in temperate regions. Evidence from multiple co-distributed troglophilic species would further strengthen this hypothesis and help to discriminate among common patterns and idiosyncratic (e.g., species-specific) patterns, which might be the case with *P. reddelli*.

### Phylogeography of Edwards Plateau karst species

Our study adds to a small number of phylogeographical studies on Edwards Plateau karst species. Much of this research has focused on terrestrial and aquatic salamanders [94-97], some of which are cave-obligate (troglobiotic) species. The phylogeography of invertebrates such as troglophilic cave crickets [98] and troglomorphic cave spiders [88,99] has also been studied. These studies revealed a pervasive pattern of relatively high genetic divergence and considerable phylogeographical structuring within or among species. The depth of phylogeographical structure includes relatively deep divergences (cave spiders: most pairwise divergences between taxa > 5%), moderate divergences (terrestrial and aquatic salamanders: most pairwise divergences around 3–5%), and relatively shallow divergences (cave crickets: most pairwise divergences < 3%). *Pseudouroctonus reddelli* appears to be at the lower end of this range with average mtDNA pairwise divergences between localities ranging from 2.4% (New Braunfels/Junction) to 1% (Camp Wood/Kickapoo). Taylor *et al.*[98] noted genetic discontinuities near the Colorado River in several karst species, including cave crickets, troglobiotic harvestmen, aquatic isopods, and terrestrial and aquatic salamanders. Most *P. reddelli* from north of the Colorado River (Austin, Georgetown, and Thor’s Cave localities) also form a distinct group in the mtDNA tree (subclade 2 in Figure 2), indicating that the Colorado River is probably a barrier to dispersal for many karst species, as previously suggested [98].

### Implications for cave conservation

Cave systems often harbour endemic troglobiotic species and frequently receive special legal protection. However, caves in temperate regions should be further prioritized for conservation because many serve as microrefugia for troglophilic species. Caves provide relatively stable microclimates during times of climate change and can facilitate persistence of troglophilic species even as their regional epigean populations diminish. As favourable environmental conditions reappear, expansion from cave microrefugia may allow some populations to respond rapidly to climate change [12]. Accordingly caves can potentially ameliorate the negative effects of anthropogenic barriers to dispersal. Caves serving as microrefugia appear to have been important in the formation and maintenance of epigean populations in temperate karst regions and are likely to continue to be important for their future survival in response to climate change.

## Conclusions

Our findings support the hypothesis that caves served as microrefugia for *P. reddelli*. The ability of this scorpion to exploit both epigean and hypogean habitats probably allowed it to persist across its present distribution during Pleistocene climatic fluctuations. Similar patterns of genetic structure in *P. reddelli* and other troglophilic species across the Edwards Plateau karst region of Texas suggest that caves serving as microrefugia are important for the formation, maintenance, and future survival of troglophilic species in temperate karst regions.

### Availability of supporting data

Sequence data used in this study have been deposited in GenBank (KF982915–KF983014) and the Dryad Digital Repository (http://dx.doi.org/10.5061/dryad.66rd8).

## Competing interests

The authors declare that they have no competing interests.

## Authors’ contributions

RWB, LP, WES, and PBP designed the research; RWB and LP acquired the samples; RWB and LP obtained the genetic data; RWB and PBP analysed the data; RWB wrote the manuscript with contributions from all authors, who read and approved the final manuscript.

## Supplementary Material

Additional file 1 **Correlations, based on Pearson’s correlation coefficient (*r*), between diversity indices (haplotype diversity, *H***_***d***_**, and nucleotide diversity, π) calculated from mitochondrial DNA (mtDNA) and nuclear internal transcribed spacer region (ITS) DNA sequence data, and latitude, longitude, and sample size for localities of the North American vaejovid scorpion *Pseudouroctonus reddelli*.** Significant correlations (*P* < 0.5) indicated in boldface. Click here for file

Additional file 2 **Network of phased internal transcribed spacer region (ITS) alleles from 43 samples of the North American vaejovid scorpion *Pseudouroctonus reddelli*.** Click here for file

Additional file 3 **Bayesian skyride plots inferred from 1,152 base pairs of mitochondrial DNA showing change in effective population size of females in two strongly supported subclades of the North American vaejovid scorpion *Pseudouroctonus reddelli *** **since the time of the most recent common ancestor.** HPD = highest posterior density; LGM = Last Glacial Maximum. Click here for file
