# Supplementary material for: Caves as microrefugia: Pleistocene phylogeography of the troglophilic North American scorpion Pseudouroctonus reddelli
Source: BMC Evol Biol. 2014 Jan 16;14:9. doi: 10.1186/1471-2148-14-9 (PMC3902065; doi:10.1186/1471-2148-14-9)
Supplement: Additional file 1 — Correlations, based on Pearson’s correlation coefficient ( r ), between diversity indices (haplotype diversity, H d , and nucleotide diversity, π) calculated from mitochondrial DNA (mtDNA) and nuclear internal transcribed spacer region (ITS) DNA sequence data, and latitude, longitude, and sample size for localities of the North American vaejovid scorpion Pseudouroctonus reddelli . Significant correlations (P < 0.5) indicated in boldface. [file 1471-2148-14-9-S1.docx]

**Additional file 1.** Correlations, based on Pearson’s correlation coefficient (*r*), between diversity indices (haplotype diversity, *H_d_*; and nucleotide diversity, π) calculated from mitochondrial DNA (mtDNA) and nuclear internal transcribed spacer region (ITS) DNA sequence data, and latitude, longitude, and sample size for localities of the North American vaejovid scorpion *Pseudouroctonus reddelli*. Significant correlations (*P* < 0.5) indicated in boldface.

|  | Latitude | | Longitude | | Sample size | |
| --- | --- | --- | --- | --- | --- | --- |
|  | *Hd* | π | *Hd* | π | *Hd* | π |
| mtDNA | ***r* = -0.6718**  ***P* = 0.0475** | *r* = -0.2404  *P* = 0.5333 | *r* = 0.1862  *P* = 0.6315 | *r* = 0.3096  *P* = 0.4175 | *r* = 0.4371  *P* = 0.2394 | *r* = 0.5897  *P* = 0.095 |
| ITS | ***r* = -0.7603**  ***P* = 0.0174** | *r* = -0.1297  *P* = 0.7395 | *r* = 0.1341  *P* = 0.7308 | *r* = 0.2323  *P* = 0.5476 | *r* = 0.3582  *P* = 0.3439 | *r* = 0.2249  *P* = 0.5606 |
