# Supplementary material for: Caves as microrefugia: Pleistocene phylogeography of the troglophilic North American scorpion Pseudouroctonus reddelli
Source: BMC Evol Biol. 2014 Jan 16;14:9. doi: 10.1186/1471-2148-14-9 (PMC3902065; doi:10.1186/1471-2148-14-9)
Supplement: Additional file 2 — Network of phased internal transcribed spacer region (ITS) alleles from 43 samples of the North American vaejovid scorpion Pseudouroctonus reddelli . [file 1471-2148-14-9-S2.docx]

**Additional file 2.** Network of phased internal transcribed spacer region (ITS) alleles from 43 samples of the North American vaejovid scorpion *Pseudouroctonus reddelli*. We estimated genetic structure within the ITS gene region by constructing a minimum-spanning tree with HapStar v0.7 [1] and network connection data based on pairwise distances (including alternative connections) generated in ARLEQUIN. A minimum-spanning tree, although questionably accurate [2], is useful for simplifying complex networks by connecting all haplotypes in a single graph without cycles [1]. We attempted two alternative network construction approaches (statistical parsimony and median-joining), but both resulted in such a high number of ambiguous loops that visualizations were exceedingly difficult. We present our minimum-spanning tree as a simple graphical summary of allele complexity within ITS, especially when considering that most networks make a number of oversimplifying assumptions about population histories [3, 4, 5, 6].


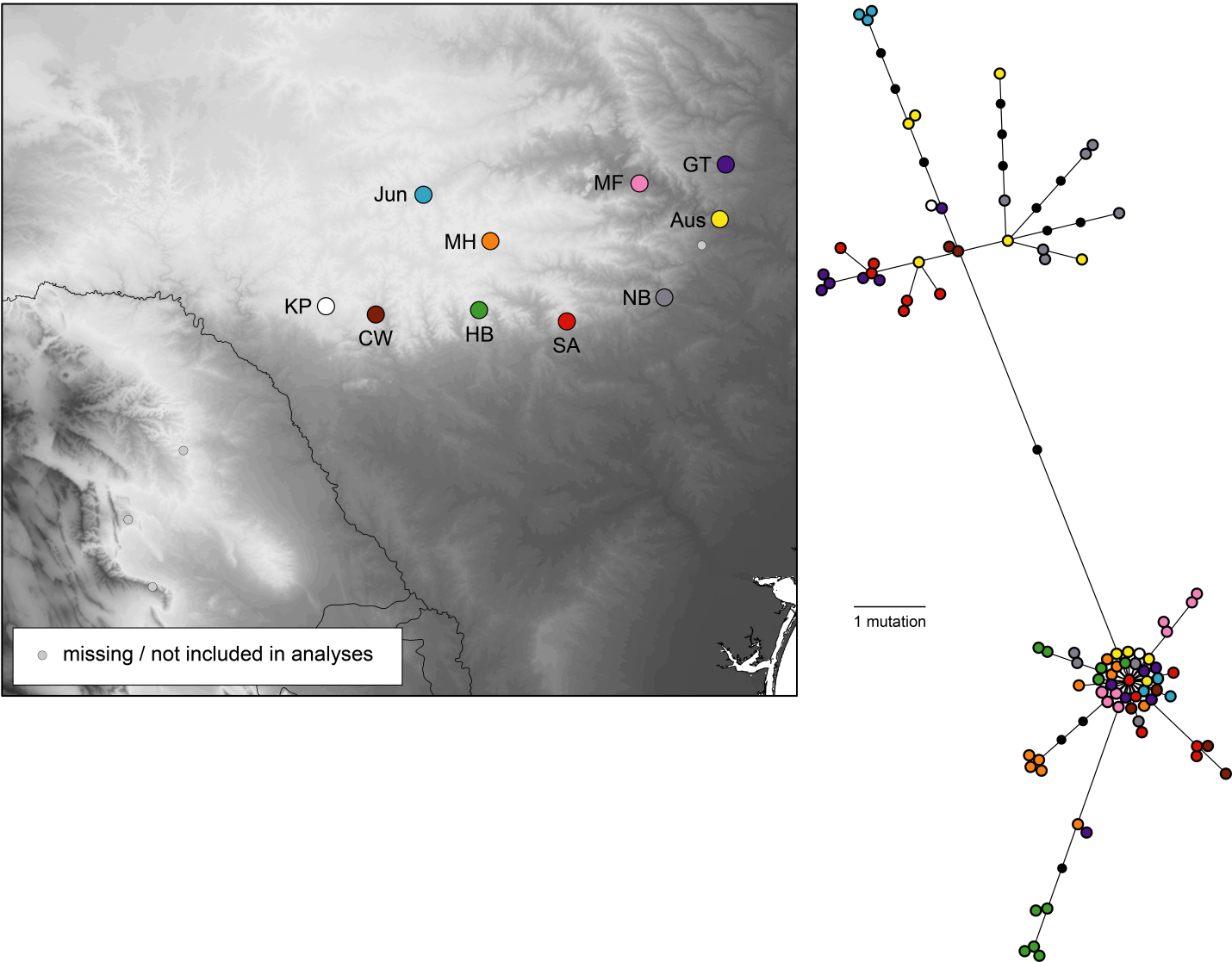


**References**

1. Teacher AGF, Griffiths DJ: **HapStar: Automated haplotype network layout and visualisation.** *Mol Ecol* *Resour* 2011, **11**:151-153.
2. Woolley SM, Posada D, Crandall KA: **A comparison of phylogenetic network methods using computer simulation.** *PLoS ONE* 2008, **3**:e1913.
3. Knowles LL, Maddison WP: **Statistical phylogeography.** *Mol Ecol* 2002, **11**:2623-2635.
4. Nielsen R, Beaumont MA: **Statistical inferences in phylogeography.** *Mol Ecol* 2009, **18**:1034-1047.
5. Salzburger W, Ewing GB, von Haeseler A: **The performance of phylogenetic algorithms in estimating haplotype genealogies with migration.** *Mol Ecol* 2011, **20**:1952-1963.
6. Marduyyn P: **Trees and/or networks to display intraspecific DNA sequence variation?.** *Mol Ecol* 2012, **21**:3385-3390.
