# Supplementary figures and images for: Caves as microrefugia: Pleistocene phylogeography of the troglophilic North American scorpion Pseudouroctonus reddelli
Source: BMC Evol Biol. 2014 Jan 16;14:9. doi: 10.1186/1471-2148-14-9 (PMC3902065; doi:10.1186/1471-2148-14-9)

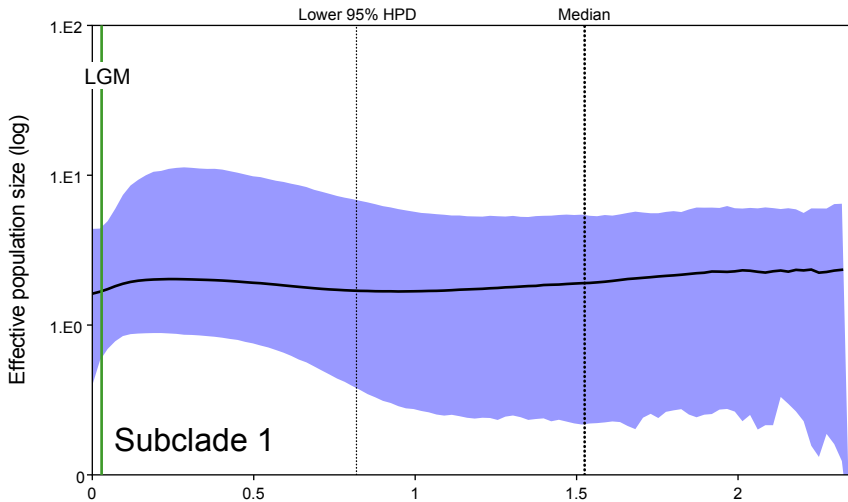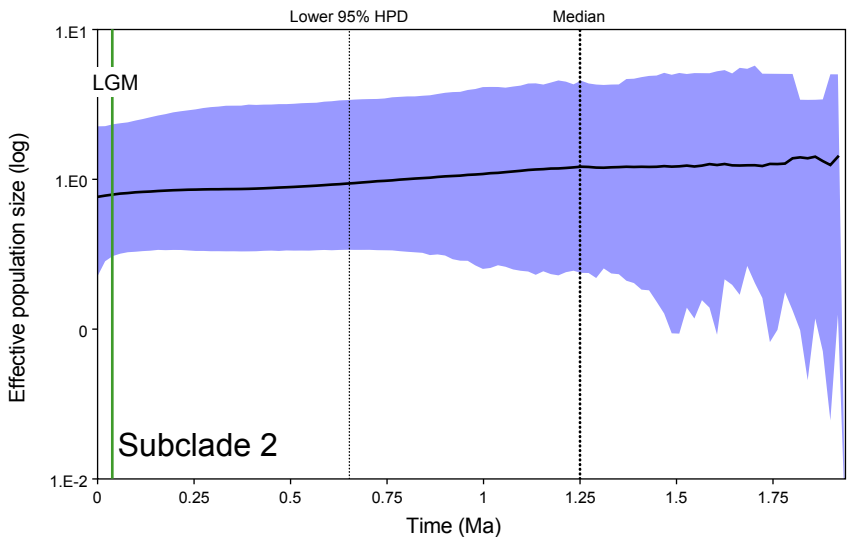

Supplement: Additional file 3 — Bayesian skyride plots inferred from 1,152 base pairs of mitochondrial DNA showing change in effective population size of females in two strongly supported subclades of the North American vaejovid scorpion Pseudouroctonus reddelli since the time of the most recent common ancestor. HPD = highest posterior density; LGM = Last Glacial Maximum. [file 1471-2148-14-9-S3.pdf]
